# Involvement of IL-9 in Th17-Associated Inflammation and Angiogenesis of Psoriasis

**DOI:** 10.1371/journal.pone.0051752

**Published:** 2013-01-15

**Authors:** Tej Pratap Singh, Michael P. Schön, Katrin Wallbrecht, Alexandra Gruber-Wackernagel, Xiao-Jing Wang, Peter Wolf

**Affiliations:** 1 Research Unit for Photodermatology, Department of Dermatology, Medical University of Graz, Graz, Austria; 2 Center for Medical Research, Medical University of Graz, Graz, Austria; 3 Department of Dermatology, Venereology and Allergology, University Medical Center Göttingen, Göttingen, Germany; 4 Departments of Pathology, Otolaryngology, Dermatology, and Craniofacial Biology, Head and Neck Cancer Research, University of Colorado Denver, Aurora, Colorado, United States of America; Universität Würzburg, Germany

## Abstract

It is thought that a Th1/Th17-weighted immune response plays a predominant role in the pathogenesis of psoriasis. Our findings now indicate a link between IL-9, a Th2 and Th9 cytokine, and Th17 pathway in psoriasis. In K5.hTGF-β1 transgenic mice, exhibiting a psoriasis-like phenotype, we found increased IL-9R and IL-9 expression in the skin and intradermal IL-9 injection induced Th17-related inflammation. IL-9 also promoted angiogenesis and VEGF and CD31 overexpression in mice *in vivo* and increased tube formation of human endothelial cells *in vitro*. Injecting anti-IL-9 antibody into K5.hTGF-β1 transgenic mice not only diminished inflammation (including skin infiltration by T cells, monocytes/macrophages, and mast cells) and angiogenesis but also delayed the psoriasis-like skin phenotype. Notably, injection of anti-psoriatic acting anti-IL-17 antibody reduced skin IL-9 mRNA and serum IL-9 protein levels in K5.hTGF-β1 transgenic mice and prevented IL-9-induced epidermal hyperplasia and inflammation of the skin of wild type mice. In addition, we observed that IL-9R expression in lesional skin from psoriasis patients was markedly higher than in healthy skin from control subjects. Moreover, IL-9 significantly enhanced IL-17A production by cultured human peripheral blood mononuclear cells or CD4+ T cells, especially in psoriasis patients. Thus, IL-9 may play a role in the development of psoriatic lesions through Th17-associated inflammation and angiogenesis.

## Introduction

Interleukin (IL)-9, a member of the IL-2 cytokine family [1], is secreted by naïve CD4+ T cells in response to TGF-β and IL-4 (Th9 pathway) [2]. However, IL-9 is also produced by activated Th2 lymphocytes and is involved in Th2-associated diseases [3]–[6]. Moreover, IL-9 is a growth factor for mast cells and T cells that help facilitate the Th9 immune response of allergic inflammatory diseases including asthma [3]–[4], [6]–[8]. The differentiation of Th9 and Th2 cells seems to be regulated by different transcription factors depending upon the cytokine environment [9]–[11]. Intriguingly, IL-9 can also induce Th17 cells to differentiate and mediate autoimmune and inflammatory diseases [3], [12]–[14]. IL-9 is also produced by Th17 cells, which secrete mainly IL-17A and IL-17F [2], [11]. When administered alone or with IL-6 and TGF-β1, IL-9 greatly enhances the production of IL- 17 from Th17 cells *in vitro* [2], [15]. Together these observations and the location of IL-9 gene on chromosome 5 (5q31.1) [3], a psoriasis susceptibility region (5q31.1-q33.1) [16] **(Figure S1)**, prompted us to investigate the pathogenic role of IL-9 in psoriasis.

Psoriasis is one of the most common chronic inflammatory skin disorders characterized by hyperplastic epidermis with hyperkeratosis, infiltration of the dermis with inflammatory cells including T cells, macrophages, and mast cells, and increased angiogenesis with an underlying Th1/Th17-dominated immune response [17]–[20]. To address the potential role of IL-9 in psoriasis we utilized both K5.hTGF-β1 transgenic mice, which exhibit a phenotype similar to human psoriasis [21]–[27], and wild type (WT) mice. In addition, we studied IL-9R expression in psoriatic skin lesions and on CD4+ T cells and the effect of IL-9 on IL-17A production in cultured human peripheral blood mononuclear cells or CD4+ T cells from psoriasis patients.

## Materials and Methods

### Human Subjects

Blood was collected from patients with moderate to severe chronic plaque type psoriasis (i.e., body size area involved >10%) and healthy control volunteers at the Photodermatology Research Unit, Department of Dermatology, Medical University of Graz, Austria (clinical study protocol approval no. 18–116 ex 06/07, Ethics Committee of the Medical University of Graz). Written consent was obtained from all patients and volunteers enrolled in the study. PBMCs were isolated by using LymphoprepTM (Axis-Shield, Heidelberg, Germany) and used for CD4+ T cell isolation or fluorescence-activated cell sorting (FACS) analysis. Skin samples were taken from the psoriasis patients or healthy control subjects for hematoxylin-eosin (HE) and immunohistochemical staining and analysis.

### Mice

Hsd:ICR/CD-1R WT and K5.hTGF-β1 transgenic mice (on an Hsd:ICR/CD-1R background) were used, as previously described [22], [23]. The mice were bred at the University Medical Center, Göttingen, Germany, from which they were shipped to the Medical University of Graz, where all experiments were performed. The mice were housed in the animal facility of the Center for Medical Research, Medical University of Graz, and maintained under condition of alternating 12-h light and dark cycles, controlled temperature, and controlled humidity in facilities approved by the Austrian Government. Water and food were provided ad libitum. All procedures to which the mice were subjected were approved by the Austrian Government, Federal Ministry for Science and Research, through protocol no. BMWF-66.010/0027- II/10b/2009 and BMWF-66.010/0023-II/3b/2011. Mice were 8–10 weeks old at the start of an experiment and age- and sex-matched within each experiment.

### Skin Disease Severity Score

A specific disease severity score was used to rate the macroscopic appearance of mouse skin. In brief, each of three symptoms (erythema, infiltration, and scaling) was scored separately as 0 (not present), 1 (mild), 2 (moderate), or 3 (severe), and then the scores were summed, as previously described [22], [23]. The highest possible score was 9.

### Murine Tissue Collection

Mice were sacrificed 48 hours after the final antibody injection (at the end of a 4-week treatment period) or 24 hours after the last IL-9 injection and blood, spleen, and skin samples were collected. Approximately 1 cm^2^ of central dorsal skin per mouse was excised, fixed immediately in 4% buffered formaldehyde, processed routinely, and embedded in paraffin. In addition, fresh skin tissue was submerged in RNAlater® solution (Applied Biosystems, Foster City, CA) and stored at −70°C for mRNA analysis. Serum was frozen and stored at −70°C for further analysis.

### Antibodies

The following anti-human antibodies were used: fluorescein isothiocyanate (FITC) anti-human CD4 (clone RPA-T4) and Alexa Fluor® 647 anti-human IL-17A (clone eBio64CAP17) (eBiosciences, San Diego, CA); mouse anti-human CD3 (clone UCHT1) and mouse anti-human CD28 (clone CD28.2) (Pharmingen, San Diego, CA); and rabbit polyclonal anti-human IL-9R (Abcam, Cambridge, UK). The following anti-mouse antibodies were used: monoclonal rat anti-mouse CD68 (clone FA-11); polyclonal rabbit anti-mouse CD3; rabbit anti-mouse STAT3; FITC goat anti-rabbit IgG; rabbit polyclonal anti-mouse VEGF; and rabbit polyclonal anti-mouse CD31 (Abcam); and anti-mouse IL-17 (clone 50104) and anti-IL-9 (R&D Systems, Minneapolis, MN).

### Isolation and Analysis of CD4+ T cells

For isolation and analysis of CD4+ T cells from human PBMCS, non-CD4+ T cells were depleted by magnetic cell sorting (MACS®) using a cocktail of biotin-conjugated antibodies against CD8, CD14, CD16, CD19, CD36, CD56, CD123, TCRγδ, and CD235a as primary labeling reagent, and anti-biotin monoclonal antibodies conjugated to MicroBeads, as secondary labeling reagent (Miltenyi Biotech, Bergisch Gladbach, Germany).Isolated CD4+ T cells (1×10^5^) were cultured in 48- or 96-well plates (Nunc, Roskilde, Denmark) in the presence or absence of different combinations of rIL-6 (30 ng/mL), rTGF-β1 (3 ng/mL), and rIL-9 (20 ng/mL) for 4 days in RPMI medium (Sigma, St. Louis, MO, USA) supplemented with 10% FCS (v/v), penicillin (100 IU/mL), streptomycin (100 µg/mL), and L- glutamate (2 µm) (PAA Laboratories, Pasching, Austria); then, leukocyte-activating cocktail (BD Pharmingen) was added to the culture media for the last 4 hours. Cells were activated using plate-bound anti-CD3 (5 µg/mL) and soluble anti-CD28 (2.5 µg/mL). For intracellular staining of cytokines, cells were first stained for CD4 surface antigen, treated with Fix/Per (Fix/Per buffer; eBiosciences), and then further stained for IL-17A, according to the manufacturer’s instructions. The stained cells were then subjected to FACS analysis on a FACSCalibur flow cytometer and the data were analyzed with Flow Jo software (Tree Star Inc., Ashland, OR).

### ELISA and ELISpot Assay

Human or mouse IL-9 and IL-17 enzyme-linked immunosorbent assay (ELISA) kits (eBiosciences) were used to quantify IL-9 and IL-17 protein levels in serum or culture supernatants of PBMCs or CD4+ T cells, according to the manufacturer’s instructions. A human IFN-γ/IL-17 dual-color ELISpot assay kit (R&D Systems) was used to quantify IFN- γ-secreting, IL-17-secreting, or IFN-γ/IL-17-co-secreting CD4+ T cells.

### Real-time RT-PCR Analysis

RNA was isolated from mouse dorsal skin with a QIAGEN fibrous mini kit (QIAGEN, Valencia, CA). RNA was reverse-transcribed with a First Strand cDNA Synthesis kit (Roche) and then subjected to quantitative RT-PCR with pretested primers for STAT3, IL-17A, and IFN-gamma (Super Array Biosciences Corporation, Frederik, MD). The reactions were run on an Applied Biosystems 7900HT system in RT^2^ SYBR Green/ROX qPCR Master Mix (Super Array Biosciences Corporation). The delta-delta Ct method was used to normalize transcript levels to GAPDH levels and to calculate fold-change from transcript levels in WT control skin samples.

### *In vitro* Angiogenesis Assay

IL-9-dependent tube formation in HMVEC cells was assayed using the In Vitro Angiogenesis Assay Kit (Millipore, Billerica, MA). Total 2×10^4^ cells were seeded in ECG medium with or without 100 ng/mL human rIL-9 (eBiosciences) and then cells placed on top of ECMatrix gels in 48-well plates and incubated for 48 hours. Tube formation was assayed after 24 and 48 hours.

### Injection of rIL-9 into Mouse Skin

Murine recombinant IL-9 (eBiosciences) (500 ng) or PBS vehicle control was injected into the dorsal skin of WT or K5.hTGF-β1 transgenic mice daily for 4 days. Twenty-four hours after the last injection, mice were sacrificed and their dorsal skin was collected.

### Neutralization of *in vivo* Bioactivity of IL-9 and IL-17

Anti-IL-9 (10 mg/kg) antibody, anti-IL-17 (10 mg/kg) antibody, or isotype IgG antibody (control) was injected intraperitoneally in K5.hTGF-β1 transgenic mice twice a week for 4 weeks. This was done to assess the neutralizing effects of the antibodies on the *in vivo* bioactivity of IL-9 and IL-17.

### Histology

Paraffin-embedded tissues of human psoriatic skin and murine skin were sectioned into 4-µm slices for HE and/or Giemsa staining.

### Immunohistochemistry

Paraffin-embedded tissue sections of human psoriatic skin and healthy human skin were stained with anti-human IL-9R or anti-human IL-9. Those of dorsal mouse skin were stained with anti-mouse IL-9, anti-mouse VEGF, anti-mouse CD31, anti-mouse CD68, or anti-mouse CD3 antibody. In brief, primary antibodies were applied to sections pretreated with EDTA at pH 8. Biotinylated polyclonal rabbit anti-rat immunoglobulins or multi-link anti-goat, -mouse, or -rabbit immunoglobulins were used with the Multilink system (Dako, Glostrup, Denmark) to visualize staining, according to the manufacturer’s instructions.

### Immunofluorescent Staining of STAT3

Paraffin-embedded tissue sections of mouse dorsal skin were indirectly stained with anti-mouse rabbit STAT3. Goat anti-rabbit IgG FITC was used as secondary antibody. In brief, antibodies were applied to sections pretreated with EDTA, pH 8. Antibody was then blocked with 5% bovine serum albumin/0.5% Tween 20. After incubation at room temperature for 1 hour, slides were incubated with secondary antibody, washed, and cover-slipped with VECTASHIELD mounting medium and DAPI (Vector Laboratories, Burlingame, CA). Images were acquired by a DP71 digital camera (Olympus, Center Valley, PA) attached to an Olympus BX51 microscope. Fluorescence intensity of STAT3 was measured by cell D software (Olympus, Vienna, Austria).

### Microscopic Skin Inflammation Assessment

Epidermal hyperplasia was quantified in HE-stained sections of dorsal skin by measuring the epidermal thickness from basal layer to stratum corneum with the calibrated eyepiece micrometer of a microscope. The number of CD3+ T cells, CD68+ monocytes/macrophages, and mast cells in the dermis of dorsal skin was assessed in at least 10–15 randomly selected areas per section (final magnification, ×200). All measurements were made blinded. Results were first averaged per mouse and then averaged per treatment group for statistical analysis.

### Angiogenesis Score

Angiogenesis in the dermis was scored as 0 (none), 1 (low), 2 (medium), 3 (high), or 4 (very high) by immunohistochemical staining for VEGF or CD31 positivity.

### Statistical Analysis

Data were expressed as mean ± SEM, as indicated in the figure legends. Statistical differences among experimental groups were determined by using 2-tailed *t*-tests as appropriate. Statistical significance was set at *P*<0.05.

## Results

### IL-9 Promotes Skin Inflammation in Mice

**Figure 1:**
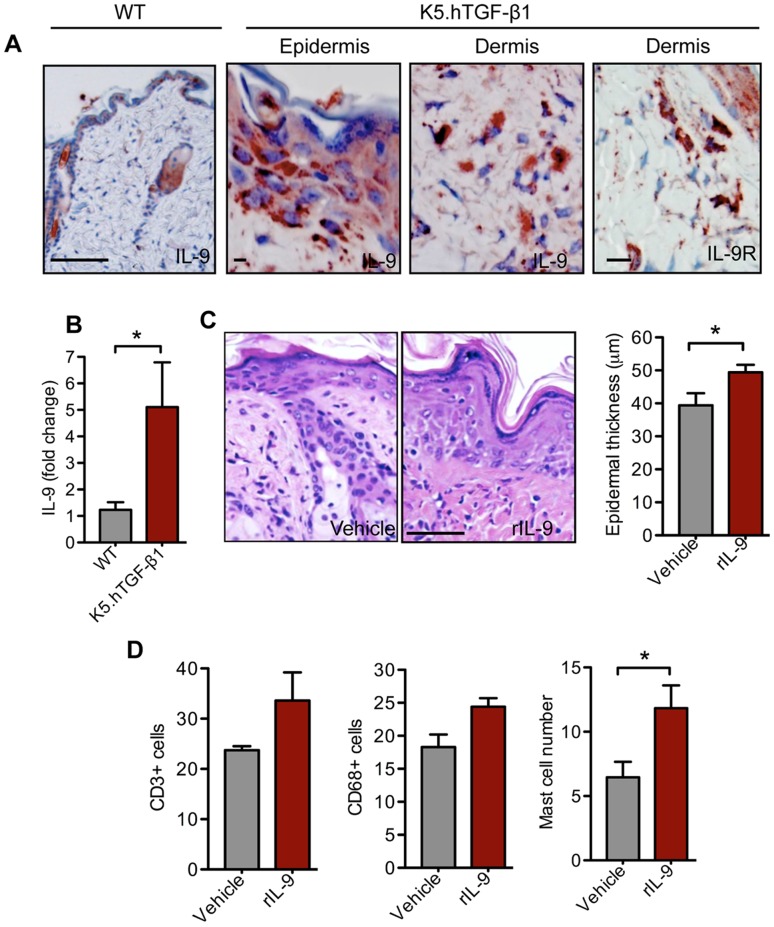
IL-9 accelerates psoriasis-like inflammation in K5.hTGF-β1 transgenic mice. (**A**) Representative photomicrographs of immunohistochemical staining of IL-9 and IL-9R in the dorsal skin of K5.hTGF-β1 transgenic mice (Scale bar 200 µm for WT and 50 and 100 µm for K5.hTGF-β1 epidermis and dermis, respectively). (**B**) Real time PCR analysis for IL-9 in the dorsal skin of WT and K5.hTGF-β1 transgenic mice (n = 7 mice per group). (**C**) K5.hTGF-β1 transgenic mice were injected intradermally for 4 days with 500 ng of IL-9 or vehicle (PBS) and skin samples were collected 24 hours after the last IL-9 injection (C,D). Representative photomicrographs of HE-stained paraffin-embedded skin sections (Scale bar 200 µm). Histological quantification of mean epidermal thickness (n = 5 mice per group). (**D**) Dermal infiltration by CD3+ T cells, CD68+ monocytes/macrophages, and mast cells in the dorsal skin of WT mice (n = 5 mice per group). Data shown represent mean numbers of cells per ×200 microscopic field. Error bars represent SEM. *, p)0.05 (unpaired t-test). Similar results were obtained in two independent experiments.

First, we evaluated IL-9 levels in K5.hTGF-β1 transgenic mice and found that IL-9 protein and mRNA expression and IL-9R protein levels were higher in the skin of the transgenic mice than in the skin of WT mice **(** **Figure 1A,B** **)**. Injecting IL-9 daily for 4 days into the skin of K5.hTGF-β1 transgenic mice enhanced epidermal hyperplasia **(** **Figure 1C** **)** and skin infiltration by CD3+ T cells, CD68+ monocytes/macrophages, and mast cells **(** **Figure 1D** **)**. The number of neutrophils did not significantly differ between the different treatment groups upon IL-9 injection (data not shown). On the other hand, treatment of the transgenic mice with anti-IL17 antibody twice a week for 4 weeks inhibited the psoriatic-like skin phenotype and downregulated IL-9 mRNA in skin and protein levels in serum (data not shown).

**Figure 2:**
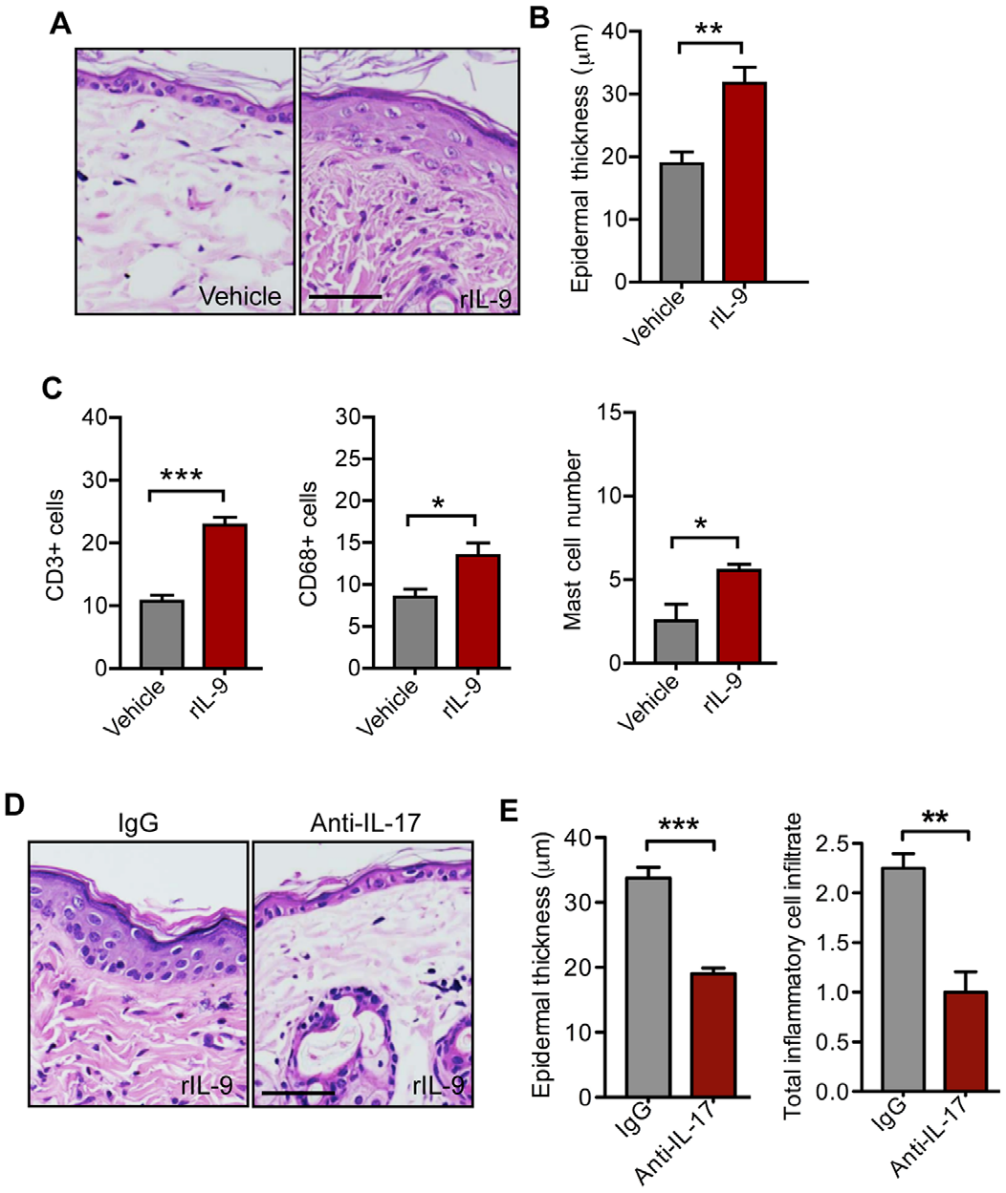
IL-9 induces inflammation in WT mice. WT mice were injected intradermally once daily for 4 days with 500 ng of recombinant IL-9 or vehicle (PBS) and skin samples were collected 24 hours after the last IL-9 injection. (**A**) Representative photomicrographs of HE-stained paraffin-embedded skin sections. (**B**) Histological quantification of mean epidermal thickness (n = 3 mice per group). (**C**) Dermal infiltration by CD3+ T cells, CD68+ monocytes/macrophages, and mast cells in the dorsal skin of WT mice (n = 3 mice per group). Data shown represent mean numbers of cells per ×200 microscopic field. (**D**) Representative photomicrographs of HE-stained paraffin-embedded skin sections of WT mice injected i.p. with either anti-IL-17 antibody or IgG isotype control antibody immediately before the first intradermal injection of 500 ng IL-9. (Scale bar 200 µm). (**E**) Histological quantification of mean epidermal thickness or semi-quantitative rating of total dermal inflammatory cell infiltrate (0, no; 1, mild; 2, moderate; and 3, severe) in each experimental group (n = 4 mice per group). Error bars represent SEM. *, p)0.05; **, p)0.01; ***, p)0.001. (unpaired t-test). Similar results were obtained in three independent experiments.

To gain further insight of IL-9’s role in psoriasis, we examined the functional role of this cytokine by giving intradermal injection of IL-9 into the back skin of WT mice. Injecting IL-9 once daily for 4 days induced skin inflammation by increasing epidermal hyperplasia **(** **Figure 2A,B** **)** and skin infiltration by CD3+ T cells, CD68+ monocytes/macrophages, and mast cells **(** **Figure 2C** **)**. We further hypothesized that IL-17 may mediate skin inflammation after IL-9 injection, given that in humans IL-9 increased Th17 differentiation [3]. Blocking IL-17 before IL-9 injection resulted in complete reversal of skin inflammation and epidermal hyperplasia **(** **Figure 2D,E** **)**.

### IL-9 Induces the Th17 Pathway in Mice

**Figure 3:**
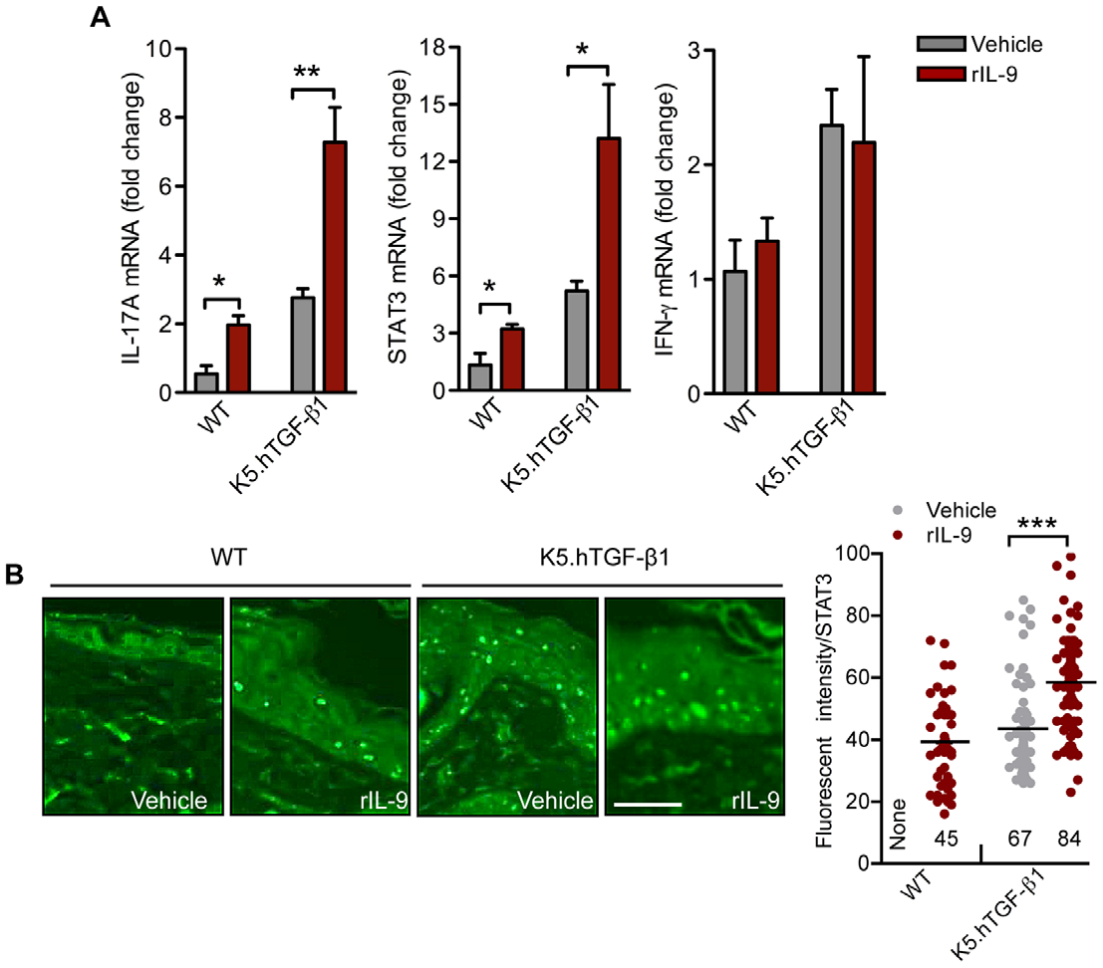
IL-9 induces Th17 pathway in both WT and K5.hTGF-β1 transgenic mice. WT or K5.hTGF-β1 transgenic mice were injected intradermally once daily for 4 days with 500 ng of IL-9 or vehicle (PBS) and tissue samples were collected 24 hours after the last injection. (**A**) Real time PCR analysis of IL-17A, STAT3 and IFN-γ in the dorsal skin of WT and K5.hTGF-β1 transgenic mice (n = 3–4 mice per group). (**B**) Representative photomicrographs of immunofluorescently stained STAT3 in the paraffin-embedded skin sections (Scale bar 200 µm). (**C**) Fluorescence intensity of STAT3 was measured in the epidermis in three randomly selected areas of the section. Numbers along the x-axis represent STAT3-positive cells. Error bars represent SEM. *, p)0.05; **, p)0.01; ***, p)0.001. (unpaired t-test). Similar results were obtained in two independent experiments.

To examine the role of Th17-cell mediated inflammation in the effects of IL-9, we analyzed the IL-9-injected skin from WT and K5.hTGF-β1 mice. We initially found and confirmed here that the transgenic mice had higher epidermal protein and total skin mRNA levels of STAT3 **(** **Figure 3A–C** **)** as well as skin IL-17 mRNA **(** **Figure 3A** **)** than the WT mice [22], [23]. Recombinant IL-9 treatment further enhanced the expression of STAT3 proteins and mRNA of STAT3 and IL-17A in both transgenic and WT mice **(** **Figure 3A–C** **).** However, when we examined the effect of IL-9 on the pro-psoriatic cytokine IFN-γ [20], we found that IL-9 had no effect on IFN-γ mRNA expression in the skin of the transgenic mice **(** **Figure 3A** **)**.

### IL-9 Induces Angiogenesis *in vitro* and *in vivo*

**Figure 4:**
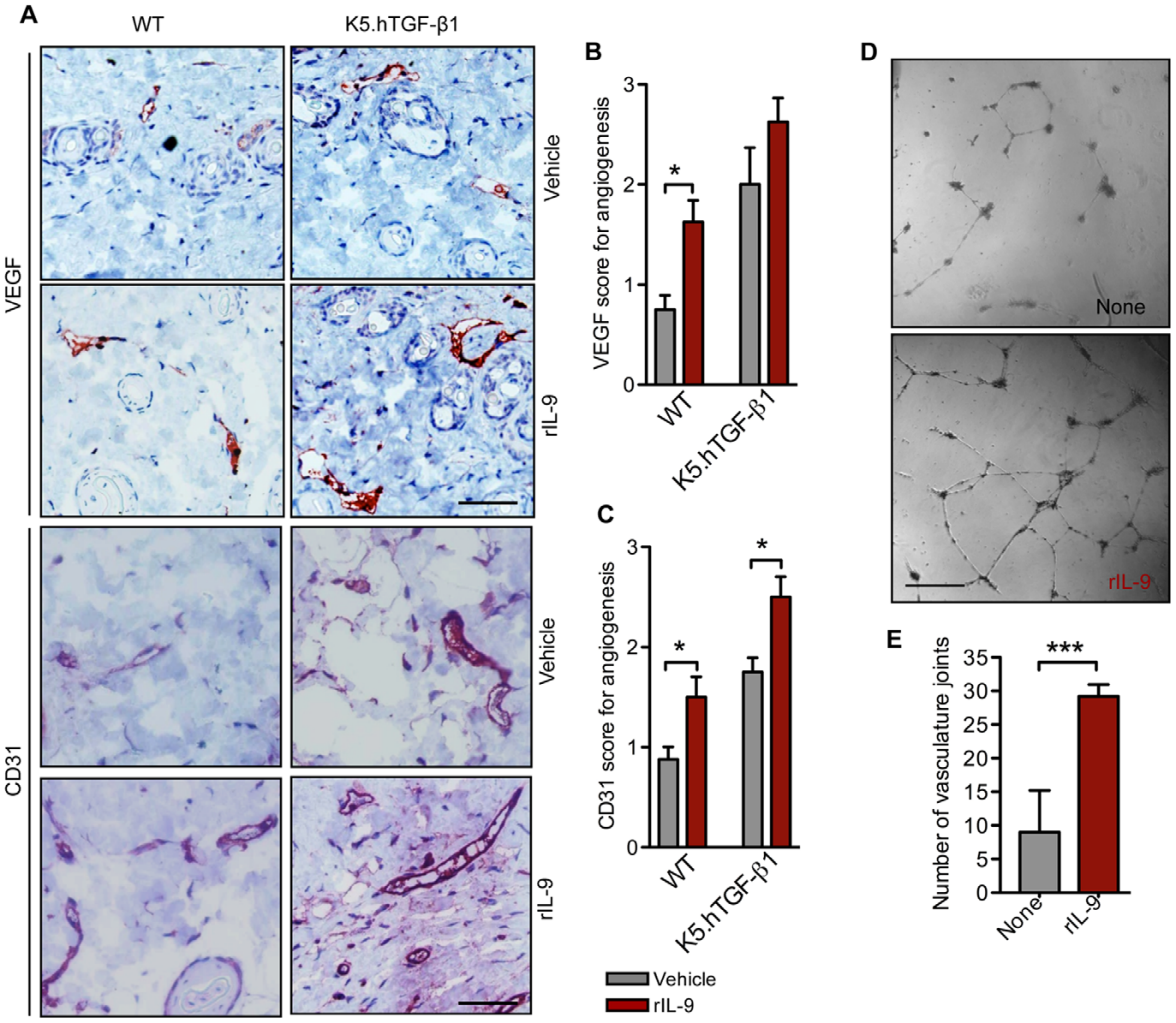
IL-9 induces angiogenesis in mice and tube formation in HDMEC. (**A**) Representative photomicrographs of VEGF and CD31 staining of paraffin-embedded sections of skin from WT or K5.hTGF-β1 transgenic mice injected intradermally for 4 days with 500 ng of IL-9 or vehicle (PBS) (Scale bar 200 µm). (**B,C**) Semi-quantitative scoring of VEGF and CD31 positivity (n = 4 mice per group). (**D**) *In vitro* angiogenesis assay (tube formation) was performed with human dermal micro vascular endothelial cells (HDMEC) in the presence or absence of IL-9 (scale bars 100 µm). (**E**) Bifurcations were counted as a measure of blood vessel formation. Data are from one of two independent experiments (n = 5 random fields in each sample). Error bars represent SEM. *, p)0.05; ***, p)0.001. (unpaired t-test). Similar results were obtained in three independent experiments.

Increased blood vessel formation and morphological and functional changes of microvessels are typical features of chronic inflammatory disorders, including psoriasis [24]. To evaluate the effect of IL-9 on blood vessel formation or angiogenesis, we injected IL-9 to the skin of WT and K5.hTGF-β1 transgenic mice. We observed that IL-9 increased the presence of angiogenic markers VEGF and CD31 in both WT and K5.hTGF-β1 transgenic mice **(** **Figure 4A–C** **)** as revealed by immunohistochemistry of the IL-9-injected skin. Next, we performed an *in vitro* angiogenesis assay with human dermal microvascular endothelial cells (HDMECs) to confirm the direct effect of IL-9 on blood vessel formation. We found that IL-9 significantly increased tube formation in HDMECs from 9.0±2.7 (baseline) to 29.2±0.8% (p<0.0001), as measured by number of vascular joints or bifurcations **(** **Figure 4D,E** **)**.

### IL-9 Neutralization Alters the Psoriatic-like Skin Inflammation and Inhibits Angiogenesis in K5.hTGF-β1 Transgenic mice

**Figure 5:**
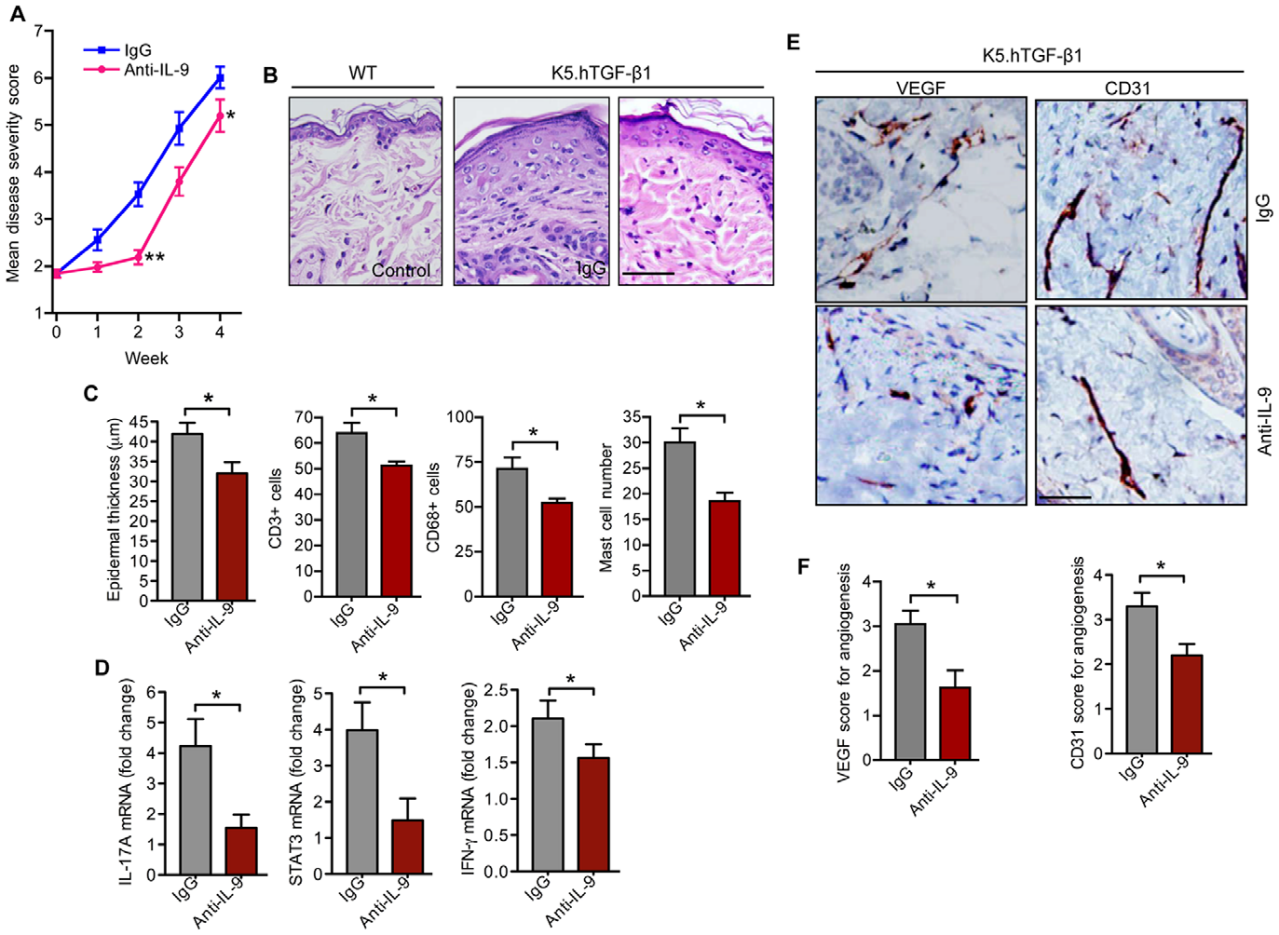
Anti-IL-9 therapy inhibits the psoriatic skin phenotype, inflammation and angiogenesis in K5.hTGF-β1 transgenic mice. K5.hTGF-β1 transgenic mice were injected i.p. with either anti-IL-9 antibody or IgG isotype control antibody (10 mg/kg) twice a week for 4 weeks and skin samples were collected at the end of week 4 for analysis (n = 5 mice per treatment group). WT mice served as controls. (**A**) Mean diseases severity scores for anti-IL9- vs. IgG-treated mice. (**B**) Representative photomicrographs of HE-stained paraffin-embedded sections of the skin from K5.hTGF-β1 transgenic mice at the end of week 4 of treatment vs. skin of an untreated WT mouse (Scale bar 200 um). (**C**) Epidermal thickness and dermal infiltration cells by CD3+ T cells, CD68+ monocytes/macrophages, and mast cells (mean numbers per ×200 microscopic field) in the dorsal skin of K5.hTGF-β1 transgenic mice. (**D**) Real-time PCR analysis of IL-17A, STAT3, and IFN-γ in the dorsal skin of K5.hTGF-β1 transgenic mice. (**E**) Representative photomicrographs of VEGF and CD31 staining of paraffin-embedded sections of skin from K5.hTGF-β1 transgenic mice (Scale bar 200 um). (**F**) Semi-quantitative scoring of VEGF and CD31 positivity. Error bars represent SEM. *, p)0.05; **, p)0.01. (Uunpaired t-test).

IL-9 neutralization has been effective in other models of autoimmune disease, including experimental autoimmune encephalitis (EAE). Anti-IL-9 treatment not only attenuated the diseases but also altered Th17 development in EAE [12], [14]. In sight of this, we neutralized the bioactivity of IL-9 in K5.hTGF-β1 transgenic mice by injecting anti-IL-9 antibody (10 mg/kg) twice a week for 4 weeks. We observed that anti-IL-9 treatment led to marked alleviation of the psoriatic phenotype in K5.hTGF-β1 transgenic mice **(** **Figure 5A,B** **)**. The effect on macroscopic phenotype alterations was greatest at week 2, when the mean skin severity score in anti-IL-9-treated transgenic mice was 37% lower than in IgG-treated control mice (ie, 2.2±0.3 vs. 3.5±0.6; p<0.01) **(** **Figure 5A**). Anti-IL-9 treatment in transgenic mice not only delayed the onset of macroscopic disease progression but also reduced histological changes, including epidermal hyperplasia and skin infiltration by CD3+ T cells, CD68+ monocytes/macrophages, and mast cells **(** **Figure 5B,C** **and Figure S2)**. The number of neutrophils did not significantly differ between the different groups upon anti-IL-9 treatment (data not shown).

To assess the immune response in skin, qRT-PCR on skin samples from K5.hTGF-β1 transgenic mice either treated with control IgG or anti-IL-9 antibody was performed. Anti-IL-9 treatment reduced the mRNA expression of IL-17A and STAT3 in the transgenic mice **(** **Figure 5D** **)**. In addition, there was a trend that anti-IL-9 treatment lowered IFN-γ mRNA expression in the skin.

We found that injecting IL-9 increased angiogenesis in K5.hTGF-β1 transgenic mice and Zibert *et al*. [24] showed that halting angiogenesis in K5.hTGF-β1 transgenic mice alleviated psoriasis-like skin inflammation. Thus, we analyzed blood vessel formation in skin and soft tissue after anti-IL-9 or control IgG antibody injection after 4 weeks of treatment. Anti-IL-9 treatment reduced macroscopic blood vessels in the skin of K5.hTGF-β1 transgenic mice **(Figure S2**). Moreover, IL-9 neutralization resulted in marked reduction in the expression of VEGF and CD31 as compared to treatment with control IgG in K5.hTGF-β1 transgenic mice on the microscopic level **(** **Figure 5E,F** **)**.

### IL-9 Promotes IL-17A Production in Human Psoriasis

**Figure 6:**
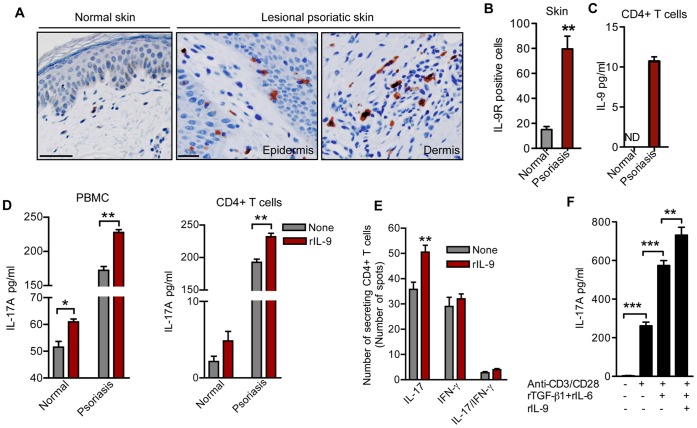
IL-9 enhances IL-17A production in human psoriasis. (**A**) Immunohistochemical staining of IL-9R in normal and lesional human psoriatic skin. (Scale bar 100 µm for normal skin and 50 µm for psoriatic skin). (**B**) Number of IL-9R positive cells in lesional skin from psoriatic patients or healthy skin from normal control subjects (n = 3 subjects per group). (**C**) Detection of IL-9 protein level in the culture supernatant of activated and cultured CD4+ T cells of healthy or psoriatic human subjects by ELISA (n = 4 subjects per group). (**D**) Detection of IL-17A in the culture supernatant of activated and cultured PBMC and CD4+ T cells of healthy or psoriatic human subjects as determined by ELISA. Cells were stimulated with IL-9 or left unstimulated (n = 3–4 subjects per group). (**E**) Dual ELIspot assay for IL-17 and IFN-γ of activated and cultured CD4+ T cells of psoriatic subjects in the presence or absence of IL-9. Number of CD4+ T cells secreting IL-17 or IFN-γ, or co-secreting IL-17 and IFN-γ were counted in samples from psoriatic patients (n = 4). (**F**) Detection of IL-17A in activated and cultured CD4+ T cells of psoriatic patients by ELISA. Cells were stimulated with different cytokine combinations (n = 4 subjects per group). Error bars represent SEM. *, p)0.05; **, p)0.01; ***, p)0.001. (unpaired t-test). ND, not detected.

To tease out the role of IL-9 in human psoriasis, we first analyzed and compared IL-9 receptor (IL-9R) expression by immunohistochemistry. We found increased IL-9R expression in scattered cells of the dermis, particularly at the dermal-epidermal junction, and within the basal layers of the epidermis in psoriatic patients compared to normal subjects **(** **Figure 6A,B** **)**. We also detected significantly higher IL-9 levels in culture supernatant of activated CD4+ T cells (10.7±0.6 pg/ml vs. ND) **(** **Figure 6C** **)**. Moreover, addition of IL-9 alone or together with IL-6 and TGF-β1 enhanced production of IL-17A by cultured and activated human psoriatic CD4+ T cells after polyclonal stimulation **(** **Figure 6F** **)**. Co-culture of CD4+T cells with IL-9, TGF-β1, and IL-6 also significantly increased IL-17A+ CD4+ T cell numbers **(Figure S3)**. Notably, IL-9-induced secretion of IL-17A and increase in IL-17A+CD4+ T cell numbers was greater for psoriasis patients than for normal healthy controls **(Figure S3)**. We also checked the effect of IL-9 on IL-17 and IFN-γ secretion by cultured CD4+ T cells isolated from human psoriatic PBMCs by ELISPOT and found increased numbers of IL-17 secreting cells but no change in the numbers of cells secreting IFN-γ or IFN-γ/IL-17 **(** **Figure 6E** **)**.

## Discussion

Our findings indicate a link between IL-9, a Th2 and Th9 cytokine, and Th17 pathway in psoriasis. We found markedly higher expression of IL- 9R in psoriatic skin lesions. In addition, we observed that *ex vivo* IL-9 stimulated the production of IL-17 by peripheral blood mononuclear cells or CD4+ T cells, especially in cells isolated from individuals with psoriasis **(** **Figure 6A–D** **)**. Accordingly, we also found that addition of IL-9 together with IL-6 and TGF-β1 increased the production of IL-17A from cultured and activated CD4+ T cells **(** **Figure 6F** **)**. Consistent with these data, Th17 cells are known to express the receptor for IL-9 [3], [11], [15]. Although, our experiments did not distinguish effects of IL-9 on the de novo production of Th17 cells from naïve cells vs. effects on effector/memory cells, earlier reports suggest that IL-9 can contribute directly to Th17 differentiations [15], and our data indicate that IL-9 may have such activity in the context of psoriasis. In addition, ELISPOT assays using cells from individuals with psoriasis showed that IL-9 had no *ex vivo* effects on numbers of IFN- γ secreting CD4+ T cells, suggesting that IL-9 makes no contribution on the Th1 component of the disease **(** **Figure 6E** **)**. This observation is also consistent with reports of IL-9′s inhibitory or null effect on IFN-γ [3], [12], [28]. Consistent with the work of others [29] we are finding that most psoriasis patients have significantly elevated IL-17 levels in the serum but intriguingly only approximately one third of them have measurable IL-9 levels (Wolf *et al*., unpublished data). However, despite normal serum levels of a cytokine such as IL-9 pathophysiologic significance may exist. Indeed, the finding that IL-9 has a pathogenic role in psoriasis is coherent with our previous observation in K5.hTGF-β1 transgenic mice, in which the therapeutic response of the psoriasiform skin to PUVA treatment correlated well with the downregulation of IL-9 in the serum [23].

Intradermal injection of IL-9 in WT mice induced local inflammation along with increased expression of IL-17A and STAT3 **(** **Figure 2** **and** **3** **)**. More support for the role of IL-9 in psoriasis comes from our findings in K5.hTGF-β1 transgenic mice. The skin of such mice is marked by hyperplastic epidermis, skin infiltration by neutrophils, T cells and macrophages, basement membrane degradation, increased angiogenesis and multiple cytokine abnormalities similar to those seen in human psoriasis [21]–[27]. Th17 cells have been implicated in this model, since the pathology can be inhibited by administering 8-methoxypsoralen plus ultraviolet A (PUVA) therapy or by blocking platelet activating factor, both of which target the Th17 pathway [22], [23]. Moreover, we have previously demonstrated that injection with anti-IL-17 antibody was able to stop progression of psoriatic disease in K5.hTGF-β1 transgenic mice [22]. We now found that there was increased IL-9R and IL-9 expression in the skin of K5.hTGF-β1 mice and intradermal IL-9 injection induced Th17-associated skin inflammation, including expression of IL-17A **(** **Figure 1** **and** **3** **)**. In addition, IL-9 may induce IL-17- or IL-22 producing γδ T cells in the skin as these cells have been recently reported to play a critical role in IL-23-induced psoriasiform dermatitis in mice [30], [31].

IL-9 has been demonstrated to play a role in models of autoimmune disease such as EAE [12], [14]. For instance, adoptive transfer of polarized Th9 cells in mice can induce the development of EAE and experimental autoimmune uveitis, through mechanisms distinct from those caused by Th1- and/or Th17-mediated inflammation. Moreover, neutralizing antibodies against IL-9 can delay the development of EAE [12], [14]. We found that injecting anti-IL-9 antibody into K5.hTGF-β1 transgenic mice not only diminished the psoriasis-like morphological changes, including cellular infiltration and neo-vascularization of the skin, but also reduced expression of IL-17A **(** **Figure 5** **)**. In addition, injecting anti-IL-17 into the K5.hTGF-β1 transgenic mice decreased skin IL-9 mRNA and serum IL-9 protein levels (data not shown). Together, these data suggest a positive feedback loop between IL-9 and IL-17A.

IL-9 is a cytokine with pleiotropic activities, including activity as a growth factor for mast cells and T cells (e. g., Th17 cells), which can secrete pro-angiogenic factors such as IL-8, IL-17, TNF, HGF, FGF-2, and VEGF [32]–[43]. Effects on these cells may have contributed to our findings that IL-9 promoted angiogenesis and VEGF and CD31 overexpression *in vivo* **(** **Figure 4** **)**. In this regard, it is of interest that we found an effect of IL-9 and anti-IL-9 treatment on mast cells, given recent evidence that mast cells may play a pathogenic role in psoriasis by augmenting VEGF release and thereby increasing inflammation via functional interactions with substance P and IL-33 [43]. In addition, we observed that treating K5.hTGF-β1 mice with anti-IL-9 inhibited blood vessel formation **(Figure S2)** and VEGF expression in skin and soft tissue **(** **Figure 5** **)**. This effect was consistent with findings from a mouse study in which anti-IL-9 treatment decreased allergen-induced lung inflammation by reducing VEGF and FGF-2 expression and mast cell numbers *in situ* [34]. However, we also provided evidence here that IL-9 has direct effects on endothelial cells to induce the formation of new vessels, since IL-9 strongly promoted tube formation by HDMEC *in vitro* **(** **Figure 4D,E** **)**. Our recent demonstration [24] that non-viral anti-angiogenic gene therapy alleviated the psoriasis-like phenotype in K5.hTGF-β1 mice (at least by part through downregulation of CD31 expression) indicates that the pro-angiogenic activity of IL-9 may be an important component in the role of this cytokine in psoriasis. The potential importance of angiogenesis as a target of anti-psoriatic treatment is consistent with recent reports, indicating that anti-VEGF treatment with monoclonal antibodies such as bevacizumab can lead to remission of psoriasis [**44**], [**45**].

Taken together, our data suggest that IL-9 has a role in the development of psoriatic lesions through Th17-associated inflammation and angiogenesis. Our data using the K5.hTGF-β1 mice also suggest that psoriasis-like inflammation can be ameliorated by anti-IL-9 treatment. This raises the possibility that similar than targeting cytokines such as TNF-α [46], IL-12/23 [47], [48], IL-17 [49]–[51], or IL-21 [52] blocking of IL-9 might be of potential benefit in patients with psoriasis and other Th17 cell-mediated autoimmune diseases.

## Supporting Information

Figure S1 Schematic representation of the genes present around IL-9 within the 5q31.1 region. The region is lying within psoriasis susceptibility 11. Search made by NCBI online Mendelian Inheritance in Man (OMIM).
(DOC)

Figure S2 Anti IL-9 treatment reduces inflammatory cell infiltration of the skin and angiogenesis in K5.hTGF-β1 transgenic mice. K5.hTGF-β1 transgenic mice were injected i.p. with either anti-IL-9 antibody or IgG isotype control antibody (n = 5 mice per group) (10 mg/kg) twice a week for 4 weeks and skin samples were collected at the end of week 4 for analysis. WT mice served as controls. **(A)** Immunohistochemical staining of CD3+ T cells and CD68+ monocytes/macrophages and Giemsa staining of mast cells. **(B, C)** The skin and adjacent soft tissue of the trunk was prepared for taking photographs from the reverse site in order evaluate the presence of blood vessels. **(B)** Example shown is from IgG isotype control antibody-injected K5.hTGF-β1 transgenic mouse, exhibiting increased angiogenesis. **(C)** Images shown are details from the periaxillary region (upper panel) and middle of the dorsum (lower panel) of the different treatment groups.
(DOC)

Figure S3 IL-9 increases the level of IL-17A+CD4+ T cells in humans. Percent of IL-17A expressing in (anti-CD3/CD28) activated and cultured human CD4+ T cells, isolated from PBMC of **(A)** normal human subjects and **(B)** psoriasis patients, as assessed by flow cytometry. Cells were stimulated either with rIL-9 alone or together with IL-6 and TGF-β1 or left unstimulated. Data represents pool of cells from one experiment with n = 3 subjects per group.
(DOC)

## References

[1] Dardalhon V, Awasthi A, Kwon H, Galileos G, Gao W, et al (2008) IL-4 inhibits TGF-beta-induced Foxp3+ T cells and, together with TGF-beta, generates IL-9+ IL-10+ Foxp3(-) effector T cells. Nat Immunol 9: 1347–1355. 18997793 10.1038/ni.1677 PMC2999006

[2] Beriou G, Bradshaw EM, Lozano E, Costantino CM, Hastings WD, et al (2010) TGF-beta induces IL-9 production from human Th17 cells. J Immunol 185: 46–54. 20498357 10.4049/jimmunol.1000356 PMC2936106

[3] Goswami R, Kaplan MH (2011) A brief history of IL-9. J Immunol 186: 3283–3288. 21368237 10.4049/jimmunol.1003049 PMC3074408

[4] Angkasekwinai P, Chang SH, Thapa M, Watarai H, Dong C (2010) Regulation of IL-9 expression by IL-25 signaling. Nat Immunol 11: 250–256. 20154671 10.1038/ni.1846 PMC2827302

[5] Steenwinckel V, Louahed J, Orabona C, Huaux F, Warnier G, et al (2007) IL-13 mediates in vivo IL-9 activities on lung epithelial cells but not on hematopoietic cells. J Immunol 178: 3244–3251. 17312173 10.4049/jimmunol.178.5.3244

[6] Hauber HP, Bergeron C, Hamid Q (2004) IL-9 in allergic inflammation. Int Arch Allergy Immunol 134: 79–87. 15133304 10.1159/000078384

[7] Lu LF, Lind EF, Gondek DC, Bennett KA, Gleeson MW, et al (2006) Mast cells are essential intermediaries in regulatory T-cell tolerance. Nature 442: 997–1002. 16921386 10.1038/nature05010

[8] Cheng G, Arima M, Honda K, Hirata H, Eda F, et al (2002) Anti-interleukin-9 antibody treatment inhibits airway inflammation and hyperreactivity in mouse asthma model. Am J Respir Crit Care Med 166: 409–416. 12153980 10.1164/rccm.2105079

[9] Chang HC, Sehra S, Goswami R, Yao W, Yu Q, et al (2010) The transcription factor PU.1 is required for the development of IL-9-producing T cells and allergic inflammation. Nat Immunol 11: 527–534. 20431622 10.1038/ni.1867 PMC3136246

[10] Staudt V, Bothur E, Klein M, Lingnau K, Reuter S, et al (2010) Interferon-regulatory factor 4 is essential for the developmental program of T helper 9 cells. Immunity 33: 192–202. 20674401 10.1016/j.immuni.2010.07.014

[11] Noelle RJ, Nowak EC (2010) Cellular sources and immune functions of interleukin-9. Nat Rev Immunol 10: 683–687. 20847745 10.1038/nri2848 PMC3828627

[12] Nowak EC, Weaver CT, Turner H, Begum-Haque S, Becher B, et al (2009) IL-9 as a mediator of Th17-driven inflammatory disease. J Exp Med 206: 1653–1660. 19596803 10.1084/jem.20090246 PMC2722185

[13] Jager A, Dardalhon V, Sobel RA, Bettelli E, Kuchroo VK (2009) Th1, Th17, and Th9 effector cells induce experimental autoimmune encephalomyelitis with different pathological phenotypes. J Immunol 183: 7169–7177. 19890056 10.4049/jimmunol.0901906 PMC2921715

[14] Li H, Nourbakhsh B, Ciric B, Zhang GX, Rostami A (2010) Neutralization of IL-9 ameliorates experimental autoimmune encephalomyelitis by decreasing the effector T cell population. J Immunol 185: 4095–4100. 20805418 10.4049/jimmunol.1000986 PMC2978501

[15] Elyaman W, Bradshaw EM, Uyttenhove C, Dardalhon V, Awasthi A, et al (2009) IL-9 induces differentiation of TH17 cells and enhances function of FoxP3+ natural regulatory T cells. Proc Natl Acad Sci U S A 106: 12885–12890. 19433802 10.1073/pnas.0812530106 PMC2722314

[16] Friberg C, Bjorck K, Nilsson S, Inerot A, Wahlstrom J, et al (2006) Analysis of chromosome 5q31–32 and psoriasis: confirmation of a susceptibility locus but no association with SNPs within SLC22A4 and SLC22A5. J Invest Dermatol 126: 998–1002. 16484987 10.1038/sj.jid.5700194

[17] Schön MP, Boehncke WH (2005) Psoriasis. N Engl J Med 352: 1899–1912. 15872205 10.1056/NEJMra041320

[18] Pittelkow MR (2005) Psoriasis: more than skin deep. Nat Med 11: 17–18. 15635435 10.1038/nm0105-17

[19] Nickoloff BJ, Wrone-Smith T (1997) Animal models of psoriasis. Nat Med 3: 475–476. 9142104 10.1038/nm0597-475b

[20] Nickoloff BJ (2007) Cracking the cytokine code in psoriasis. Nat Med 13: 242–244. 17342112 10.1038/nm0307-242

[21] Li AG, Wang D, Feng XH, Wang XJ (2004) Latent TGFbeta1 overexpression in keratinocytes results in a severe psoriasis-like skin disorder. EMBO J 23: 1770–1781. 15057277 10.1038/sj.emboj.7600183 PMC394237

[22] Singh TP, Huettner B, Koefeler H, Mayer G, Bambach I, et al (2011) Platelet-activating factor blockade inhibits the T-helper type 17 cell pathway and suppresses psoriasis-like skin disease in K5.hTGF-beta 1 transgenic mice. Am J Pathol 178: 699–708. 21281802 10.1016/j.ajpath.2010.10.008 PMC3070583

[23] Singh TP, Schon MP, Wallbrecht K, Michaelis K, Rinner B, et al (2010) 8-methoxypsoralen plus ultraviolet A therapy acts via inhibition of the IL-23/Th17 axis and induction of Foxp3+ regulatory T cells involving CTLA4 signaling in a psoriasis-like skin disorder. J Immunol 184: 7257–7267. 20488788 10.4049/jimmunol.0903719

[24] Zibert JR, Wallbrecht K, Schon M, Mir LM, Jacobsen GK, et al (2011) Halting angiogenesis by non-viral somatic gene therapy alleviates psoriasis and murine psoriasiform skin lesions. J Clin Invest 121: 410–421. 21135506 10.1172/JCI41295 PMC3007133

[25] Han G, Li F, Singh TP, Wolf P, Wang XJ (2012) The pro-inflammatory role of TGFbeta1: a paradox? Int J Biol Sci 8: 228–235. 22253566 10.7150/ijbs.8.228 PMC3258562

[26] Singh TP, Schon MP, Wallbrecht K, Wolf P (2012) 8-Methoxypsoralen plus UVA treatment increases the proportion of CLA+ CD25+ CD4+ T cells in lymph nodes of K5.hTGFbeta1 transgenic mice. Exp Dermatol 21: 228–230. 22379972 10.1111/j.1600-0625.2011.01437.x

[27] Swindell WR, Johnston A, Carbajal S, Han G, Wohn C, et al (2011) Genome-wide expression profiling of five mouse models identifies similarities and differences with human psoriasis. PLoS One 6: e18266. 21483750 10.1371/journal.pone.0018266 PMC3070727

[28] Schmitt E, Germann T, Goedert S, Hoehn P, Huels C, et al (1994) IL-9 production of naive CD4+ T cells depends on IL-2, is synergistically enhanced by a combination of TGF-beta and IL-4, and is inhibited by IFN-gamma. J Immunol 153: 3989–3996. 7930607

[29] Coimbra S, Oliveira H, Reis F, Belo L, Rocha S, et al (2010) Interleukin (IL)-22, IL-17, IL-23, IL-8, vascular endothelial growth factor and tumour necrosis factor-alpha levels in patients with psoriasis before, during and after psoralen-ultraviolet A and narrowband ultraviolet B therapy. Br J Dermatol 163: 1282–1290. 20716219 10.1111/j.1365-2133.2010.09992.x

[30] Mabuchi T, Takekoshi T, Hwang ST (2011) Epidermal CCR6+ gammadelta T cells are major producers of IL-22 and IL-17 in a murine model of psoriasiform dermatitis. J Immunol 187: 5026–5031. 21984702 10.4049/jimmunol.1101817

[31] Cai Y, Shen X, Ding C, Qi C, Li K, et al (2011) Pivotal role of dermal IL-17-producing gammadelta T cells in skin inflammation. Immunity 35: 596–610. 21982596 10.1016/j.immuni.2011.08.001 PMC3205267

[32] Eller K, Wolf D, Huber JM, Metz M, Mayer G, et al (2010) IL-9 production by regulatory T cells recruits mast cells that are essential for regulatory T cell-induced immune suppression. J Immunol 186: 83–91. 21115728 10.4049/jimmunol.1001183 PMC3227733

[33] Abdelilah S, Latifa K, Esra N, Cameron L, Bouchaib L, et al (2001) Functional expression of IL-9 receptor by human neutrophils from asthmatic donors: role in IL-8 release. J Immunol 166: 2768–2774. 11160343 10.4049/jimmunol.166.4.2768

[34] Kearley J, Erjefalt JS, Andersson C, Benjamin E, Jones CP, et al.. (2010) IL-9 governs allergen-induced mast cell numbers in the lung and chronic remodeling of the airways. Am J Respir Crit Care Med. 10.1164/rccm.200909-1462OC PMC3385369 20971830

[35] Matsuzawa S, Sakashita K, Kinoshita T, Ito S, Yamashita T, et al (2003) IL-9 enhances the growth of human mast cell progenitors under stimulation with stem cell factor. J Immunol 170: 3461–3467. 12646606 10.4049/jimmunol.170.7.3461

[36] Wiener Z, Falus A, Toth S (2004) IL-9 increases the expression of several cytokines in activated mast cells, while the IL-9-induced IL-9 production is inhibited in mast cells of histamine-free transgenic mice. Cytokine 26: 122–130. 15135806 10.1016/j.cyto.2004.01.006

[37] Stassen M, Muller C, Arnold M, Hultner L, Klein-Hessling S, et al (2001) IL-9 and IL-13 production by activated mast cells is strongly enhanced in the presence of lipopolysaccharide: NF-kappa B is decisively involved in the expression of IL-9. J Immunol 166: 4391–4398. 11254693 10.4049/jimmunol.166.7.4391

[38] Asarch A, Barak O, Loo DS, Gottlieb AB (2008) Th17 cells: A new paradigm for cutaneous inflammation. J Dermatol Treatment 19: 259–266. 10.1080/09546630802206686 18629676

[39] Chua RA, Arbiser JL (2009) The role of angiogenesis in the pathogenesis of psoriasis. Autoimmunity 42: 574–579. 19863376 10.1080/08916930903002461

[40] Halin C, Detmar M (2008) Chapter 1. Inflammation, angiogenesis, and lymphangiogenesis. Methods Enzymol 445: 1–25. 19022053 10.1016/S0076-6879(08)03001-2

[41] Heidenreich R, Röcken M, Ghoreschi K (2009) Angiogenesis drives psoriasis pathogenesis. Int J Exp Pathol 90: 232–248. 19563608 10.1111/j.1365-2613.2009.00669.x PMC2697548

[42] Simonetti O, Lucarini G, Goteri G, Zizzi A, Biagini G, et al (2006) VEGF is likely a key factor in the link between inflammation and angiogenesis in psoriasis: Results of an immunohistochemical study. Int J Immunopathol Pharmacol 19: 751–760. 17166397 10.1177/039463200601900405

[43] Theoharides TC, Zhang B, Kempuraj D, Tagen M, Vasiadi M, et al (2010) IL-33 augments substance P-induced VEGF secretion from human mast cells and is increased in psoriatic skin. Proc Natl Acad Sci U S A 107: 4448–4453. 20160089 10.1073/pnas.1000803107 PMC2840132

[44] Akman A, Yilmaz E, Mutlu H, Ozdogan M (2009) Complete remission of psoriasis following bevacizumab therapy for colon cancer. Clin Exp Dermatol 34: e202–204. 19077094 10.1111/j.1365-2230.2008.02991.x

[45] Crawshaw AA, Griffiths CE, Young HS (2012) Investigational VEGF antagonists for psoriasis. Expert Opin Investig Drugs 21: 33–43. 10.1517/13543784.2012.636351 22088218

[46] Inzinger M, Heschl B, Weger W, Hofer A, Legat FJ, et al (2011) Efficacy of psoralen plus ultraviolet A therapy vs. biologics in moderate to severe chronic plaque psoriasis: retrospective data analysis of a patient registry. Br J Dermatol 165: 640–645. 21564068 10.1111/j.1365-2133.2011.10396.x

[47] Inzinger M, Weger W, Salmhofer W, Wolf P (2012) Differential Response of Chronic Plaque Psoriasis to Briakinumab vs. Ustekinumab. Acta Derm Venereol. 92: 357–358. 10.2340/00015555-1243 22169986

[48] Wolf P, Weger W, Legat FJ, Posch-Fabian T, Gruber-Wackernagel A, et al (2012) Treatment with 311-nm ultraviolet B enhanced response of psoriatic lesions in ustekinumab-treated patients: a randomized intraindividual trial. Br J Dermatol 166: 147–153. 21910714 10.1111/j.1365-2133.2011.10616.x

[49] Leonardi C, Matheson R, Zachariae C, Cameron G, Li L, et al (2012) Anti-interleukin-17 monoclonal antibody ixekizumab in chronic plaque psoriasis. N Engl J Med 366: 1190–1199. 22455413 10.1056/NEJMoa1109997

[50] Papp KA, Leonardi C, Menter A, Ortonne JP, Krueger JG, et al (2012) Brodalumab, an anti-interleukin-17-receptor antibody for psoriasis. N Engl J Med 366: 1181–1189. 22455412 10.1056/NEJMoa1109017

[51] Tsoi LC, Spain SL, Knight J, Ellinghaus E, Stuart PE, et al.. (2012) Identification of 15 new psoriasis susceptibility loci highlights the role of innate immunity. Nat Genet. Online Nov 11. doi:10.1038/ng.2467. 10.1038/ng.2467 PMC3510312 23143594

[52] Costanzo A, Chimenti MS, Botti E, Caruso R, Sarra M, et al (2010) IL-21 in the pathogenesis and treatment of skin diseases. J Dermatol Sci 60: 61–66. 20888735 10.1016/j.jdermsci.2010.08.016

