# Supplementary material for: Involvement of IL-9 in Th17-Associated Inflammation and Angiogenesis of Psoriasis
Source: PLoS One. 2013 Jan 15;8(1):e51752. doi: 10.1371/journal.pone.0051752 (PMC3546056; doi:10.1371/journal.pone.0051752)
Supplement: Figure S1 — Schematic representation of the genes present around IL-9 within the 5q31.1 region. The region is lying within psoriasis susceptibility 11. Search made by NCBI online Mendelian Inheritance in Man (OMIM). (DOC) [file pone.0051752.s001.doc]

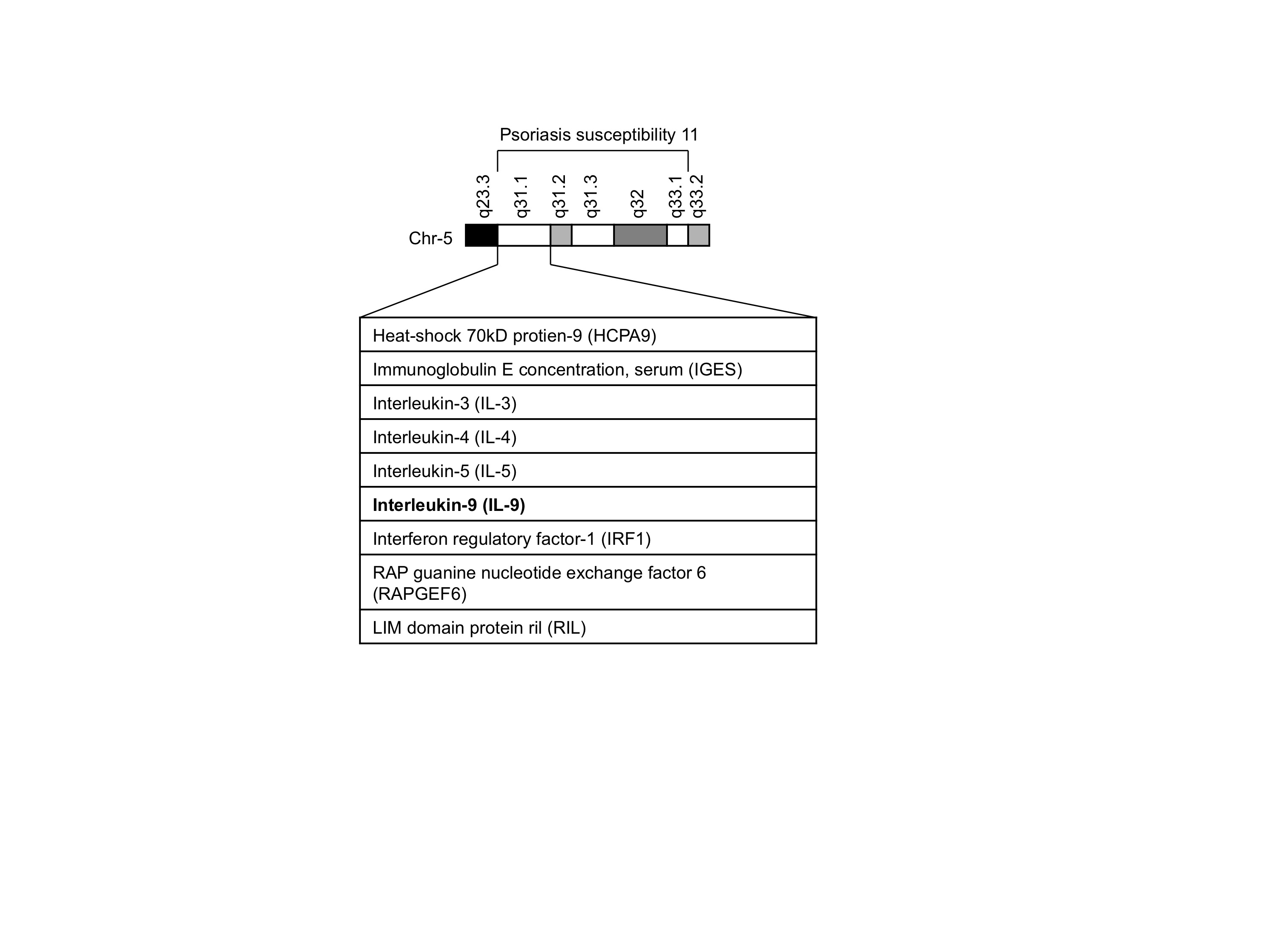


**Figure S1.** Schematic representation of the genes present around IL-9 within the 5q31.1 region. The region is lying within psoriasis susceptibility 11. Search made by NCBI online Mendelian Inheritance in Man (OMIM).
