# Supplementary material for: Involvement of IL-9 in Th17-Associated Inflammation and Angiogenesis of Psoriasis
Source: PLoS One. 2013 Jan 15;8(1):e51752. doi: 10.1371/journal.pone.0051752 (PMC3546056; doi:10.1371/journal.pone.0051752)
Supplement: Figure S2 — Anti IL-9 treatment reduces inflammatory cell infiltration of the skin and angiogenesis in K5.hTGF-β1 transgenic mice. K5.hTGF-β1 transgenic mice were injected i.p. with either anti-IL-9 antibody or IgG isotype control antibody (n = 5 mice per group) (10 mg/kg) twice a week for 4 weeks and skin samples were collected at the end of week 4 for analysis. WT mice served as controls. (A) Immunohistochemical staining of CD3+ T cells and CD68+ monocytes/macrophages and Giemsa staining of mast cells. (B, C) The skin and adjacent soft tissue of the trunk was prepared for taking photographs from the reverse site in order evaluate the presence of blood vessels. (B) Example shown is from IgG isotype control antibody-injected K5.hTGF-β1 transgenic mouse, exhibiting increased angiogenesis. (C) Images shown are details from the periaxillary region (upper panel) and middle of the dorsum (lower panel) of the different treatment groups. (DOC) [file pone.0051752.s002.doc]

**
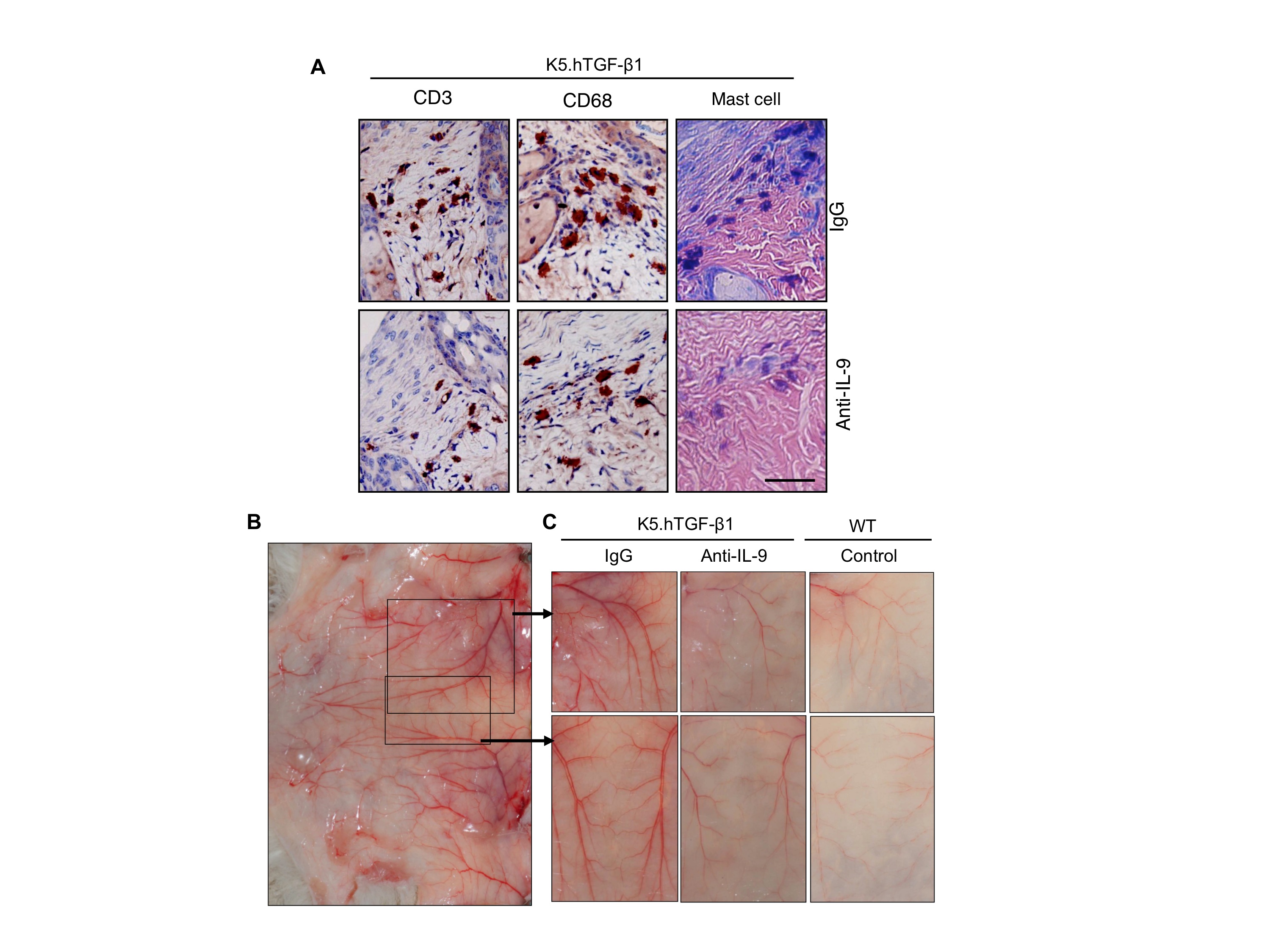
**

**Figure S2.** Anti IL-9 treatment reduces inflammatory cell infiltration of the skin and angiogenesis in K5.hTGF-β1 transgenic mice. K5.hTGF-β1 transgenic mice were injected i.p. with either anti-IL-9 antibody or IgG isotype control antibody (n=5 mice per group) (10mg/kg) twice a week for 4 weeks and skin samples were collected at the end of week 4 for analysis. WT mice served as controls. **(A)** Immunohistochemical staining of CD3+ T cells and CD68+ monocytes/macrophages and Giemsa staining of mast cells. **(B, C)** The skin and adjacent soft tissue of the trunk was prepared for taking photographs from the reverse site in order evaluate the presence of blood vessels. **(B)** Example shown is from IgG isotype control antibody-injected K5.hTGF-β1 transgenic mouse, exhibiting increased angiogenesis. **(C)** Images shown are details from the periaxillary region (upper panel) and middle of the dorsum (lower panel) of the different treatment groups.
