# Supplementary material for: Involvement of IL-9 in Th17-Associated Inflammation and Angiogenesis of Psoriasis
Source: PLoS One. 2013 Jan 15;8(1):e51752. doi: 10.1371/journal.pone.0051752 (PMC3546056; doi:10.1371/journal.pone.0051752)
Supplement: Figure S3 — IL-9 increases the level of IL-17A+CD4+ T cells in humans. Percent of IL-17A expressing in (anti-CD3/CD28) activated and cultured human CD4+ T cells, isolated from PBMC of (A) normal human subjects and (B) psoriasis patients, as assessed by flow cytometry. Cells were stimulated either with rIL-9 alone or together with IL-6 and TGF-β1 or left unstimulated. Data represents pool of cells from one experiment with n = 3 subjects per group. (DOC) [file pone.0051752.s003.doc]

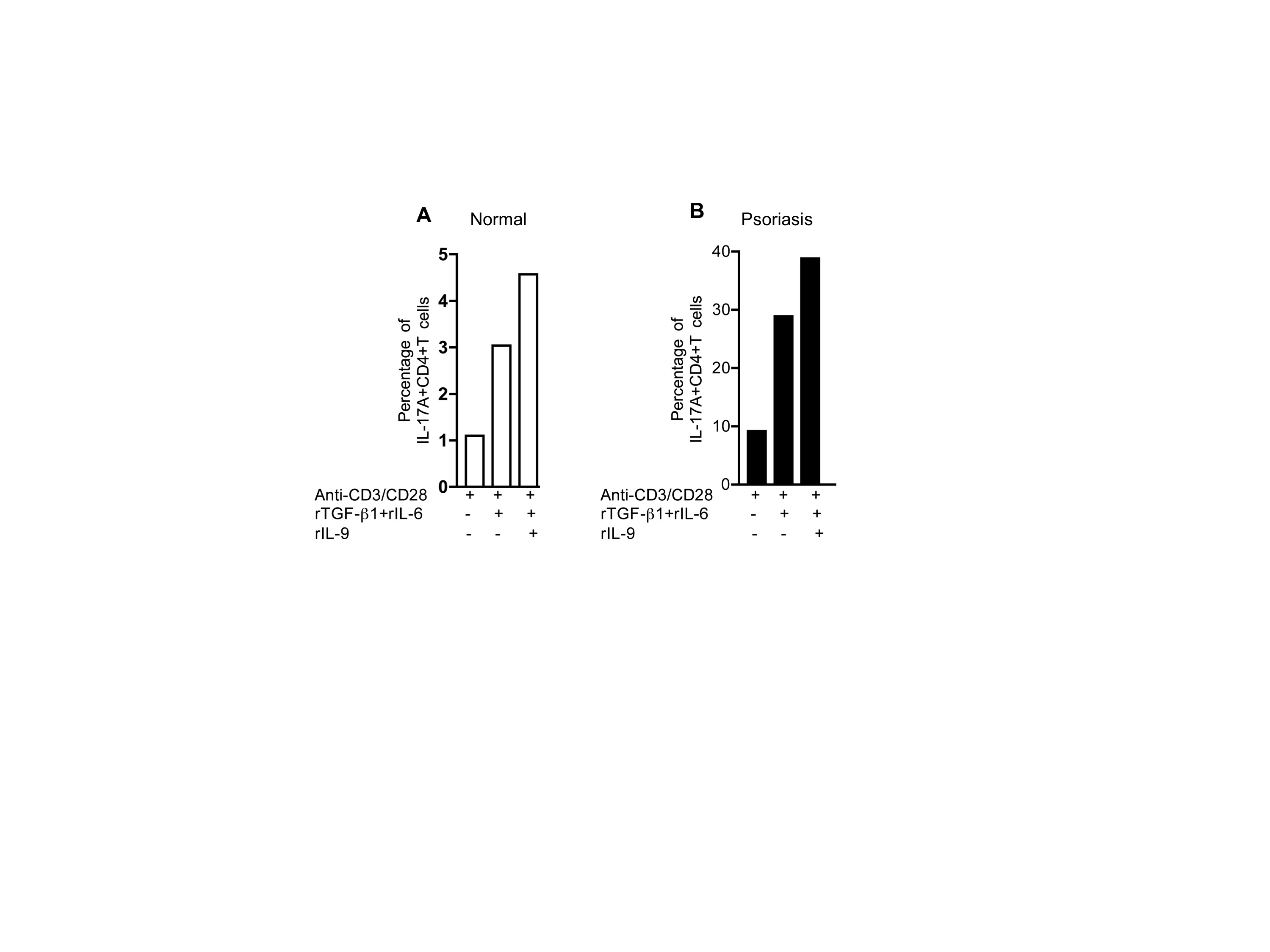


**Figure S3.** IL-9 increases the level of IL-17A+CD4+ T cells in humans. Percent of IL-17A expressing in (anti-CD3/CD28) activated and cultured human CD4+ T cells, isolated from PBMC of **(A)** normal human subjects and **(B)** psoriasis patients, as assessed by flow cytometry. Cells were stimulated either with rIL-9 alone or together with IL-6 and TGF-β1 or left unstimulated. Data represents pool of cells from one experiment with n=3 subjects per group.
